# Cocaine Directly Impairs Memory Extinction and Alters Brain DNA Methylation Dynamics in Honey Bees

**DOI:** 10.3389/fphys.2018.00079

**Published:** 2018-02-13

**Authors:** Eirik Søvik, Pauline Berthier, William P. Klare, Paul Helliwell, Edwina L. S. Buckle, Jenny A. Plath, Andrew B. Barron, Ryszard Maleszka

**Affiliations:** ^1^Department of Science and Mathematics, Volda University College, Volda, Norway; ^2^Department of Biological Sciences, Macquarie University, Sydney, NSW, Australia; ^3^Research School of Biology, Australian National University, Canberra, ACT, Australia

**Keywords:** addiction, *Apis mellifera*, DNMT3, demethylation, epigenomics, TET

## Abstract

Drug addiction is a chronic relapsing behavioral disorder. The high relapse rate has often been attributed to the perseverance of drug-associated memories due to high incentive salience of stimuli learnt under the influence of drugs. Drug addiction has also been interpreted as a memory disorder since drug associated memories are unusually enduring and some drugs, such as cocaine, interfere with neuroepigenetic machinery known to be involved in memory processing. Here we used the honey bee (an established invertebrate model for epigenomics and behavioral studies) to examine whether or not cocaine affects memory processing independently of its effect on incentive salience. Using the proboscis extension reflex training paradigm we found that cocaine strongly impairs consolidation of extinction memory. Based on correlation between the observed effect of cocaine on learning and expression of epigenetic processes, we propose that cocaine interferes with memory processing independently of incentive salience by directly altering DNA methylation dynamics. Our findings emphasize the impact of cocaine on memory systems, with relevance for understanding how cocaine can have such an enduring impact on behavior.

## Introduction

Commonly abused drugs cause debilitating drug addiction in a small fraction of users (McLellan et al., 2000). Addiction is a chronic, relapsing condition marked by compulsive drug seeking and craving (Robinson and Berridge, 2003). Recovering addicts suffer high relapse rates due to persistent drug associated memories (Hser et al., 2001). This has led some authors to conclude that drug addiction is a disease of learning and memory (Hyman, 2005; Hyman et al., 2006). Most drugs of abuse are strongly reinforcing and have high incentive salience (Siegel, 2005; Robinson and Berridge, 2008): that is, motivation to seek out drugs or drug associated cues is strong (Robinson and Berridge, 1993). Consequently, stimuli associated with drug administration are readily learned, and memories of them are persistent (Uslaner et al., 2006). It has been argued that increased incentive salience is why drug associated memories are particularly difficult to extinguish (Stewart, 2000), resulting in frequent relapses (Weiss et al., 2001). In mammalian brains, many drugs of abuse alter neurotransmission in the dopaminergic midbrain pathway either by increasing release or inhibiting clearance of dopamine (Kuhar et al., 1991; Han and Gu, 2006), thereby increasing the incentive salience of a given stimuli (Berridge, 2007).

Altered incentive salience is not the only way by which memory processing can be altered, however. Many drugs also interfere with memory processing directly (Mittenberg and Motta, 1993). Cocaine induces widespread changes in DNA methylation patterns (Anier et al., 2010). This is of particular interest, since both DNA methyltransferase enzymes (DNMT) and Ten-eleven translocation (TET) proteins, responsible for methylation and demethylation of DNA, respectively, are both vital for memory formation (Day et al., 2013; Alaghband et al., 2016; Kennedy and Sweatt, 2016). Previous studies have shown cocaine-associated memories to be correlated with changes in DNA methylation (Tian et al., 2012) and to be highly resistant to extinction (Di Ciano and Everitt, 2004). It is not yet known, however, if the enduring nature of cocaine-associated memory is due to cocaine directly affecting the DNA methylation machinery, or if it is an indirect consequence of altered incentive salience.

Honey bees have long been used to study mechanisms of associative learning (Menzel et al., 1974; Bitterman et al., 1983; Hammer and Menzel, 1995), and more recently as a valuable model system for elucidating the effects of pharmacological manipulations on learning and memory (Felsenberg et al., 2011; Maleszka, 2014). Bees have functional DNA methylation and demethylation systems (Wang et al., 2006; Lyko and Maleszka, 2011; Wojciechowski et al., 2014; Maleszka, 2016), that are involved in memory processing. Following olfactory conditioning altered methylation patterns can be seen across the entire honey bee genome (Li et al., 2017).

DNMT function is required for forming stimulus-specific olfactory memories (Biergans et al., 2012, 2016), potentially due to its activity in the antennal lobes (Biergans et al., 2017). Pharmacological inhibition of DNMTs has also been shown to interfere with consolidation extinction of appetitive memories (Lockett et al., 2010; Gong et al., 2016). *DNMT1b* and *DNMT3* and *Tet* are all upregulated following olfactory conditioning (Biergans et al., 2015), but no direct function of TET proteins during learning has been demonstrated in bees so far.

Because cocaine results in similar behavioral and neurochemical responses in bees and mammals (Barron et al., 2009; Søvik, 2013; Søvik et al., 2013, 2014), it presents itself as a valuable system to explore the basic interactions between drugs of abuse, epigenomic modifications and behavior (Søvik and Barron, 2013; Maleszka, 2014, 2016). Here we investigated the effects of cocaine on acquisition, consolidation, and retrieval of memories in honey bees when drug delivery was dissociated from conditioning, and explored whether cocaine affected brain DNA methylation systems.

## Materials and methods

### Animals

European honey bees, *Apis mellifera*, of the standard commercial strain available in New South Wales, Australia were used for all experiments. Adult bees were collected on emergence from brood cells, placed in mesh cages (20 × 16 × 3 cm) with *ad libitum* access to honey (80 bees per cage) and housed in an incubator at 34°C for 6 days prior to learning experiments. Cage rearing offers greater control of bees' age and experience it differs fundamentally from life in the hive. This can be problematic for some experiments, but as it does not significantly affect brain development (Maleszka et al., 2009) or ability to retain olfactory memories (Arenas and Farina, 2008), we decided it was the best approach for our experiments. Behavioral experiments 1–4 were conducted at The Australian National University, Canberra, while remaining experiments were conducted at Macquarie University, Sydney.

### Drug treatments

The treatments used for all experiments consisted of either 3 μg of freebase cocaine (cocaine) dissolved in 1 μL dimethylformamide (DMF) or 1 μL DMF on its own (control). All chemicals were supplied by Sigma-Aldrich (St. Louis, MO, USA). The treatments were given topically by placing 1 μL of the solution onto the dorsal thorax of bees with a microcapillary pipette. Care was taken to prevent treatments from spreading to wing joints or across the wings. DMF rapidly penetrates bee cuticle and can conduct compounds into the haemolymph of the bees' open circulatory system, from where small compounds can access the brain and nervous system (Barron et al., 2007; Okada et al., 2015). This administration method has previously been shown to be effective for delivering cocaine to honey bees (Barron et al., 2009; Søvik et al., 2013, 2016; Scheiner et al., 2014).

### Training protocols

At 6 days of age, bees were harnessed for proboscis extension response (PER) conditioning (Bitterman et al., 1983). The thorax and abdomen of bees were lightly restrained in 8 mm diameter metal tubes by a thin piece of tape placed behind the neck so the head was kept in place, but antennae and proboscis were free to move (Maleszka et al., 2000; Si, 2004; Lockett et al., 2014). Each bee was fed 2 drops (approx. 30 μL) of 1.5 M sucrose, and left overnight. On the following morning, bees were trained in either a differential (experiment 1–5), or absolute (experiment 6) conditioning paradigm. For differential conditioning bees were trained to distinguish two odors (limonene and natural vanilla), one paired to reward and the other to punishment. For absolute conditioning only a single odor associated with reward was used.

Reward training involved touching a droplet of 2 M sucrose solution to the antennae followed by offering sucrose to the proboscis. Punishment consisted of touching saturated NaCl solution to the antennae, which is strongly aversive to bees (Maleszka et al., 2000; de Brito Sanchez et al., 2005; Lockett et al., 2010, 2014). Presentation of sucrose to bees results in proboscis extension, and following paired presentation of odor and sucrose bees learn to extend their proboscis to an odor that is predictive of sucrose delivery. Following training with the aversive salt solution the proboscis is actively withheld (Smith et al., 1991). For acquisition training odors were presented for 3 s on their own, and for 2 s simultaneously with the reward/punishment. For extinction training odors were presented on their own for 5 s.

For both absolute and differential conditioning bees were given 3 learning trials, each separated by 6 min (acquisition training). In the evening bees were fed with 2 drops of 1.5 M sucrose and left overnight. On the following morning, 24 h after conditioning, bees were tested by presenting training odors alone, and whether or not bees extended their proboscis to the odors was recorded (acquisition test). For the differential conditioning, we immediately discarded all bees that were deemed non-learners after the acquisition test (i.e., responded to the odor associated with the punishment, failed to respond to the odor associated with the reward, or both). Because of this, only bees that had had the correct response (proboscis extension to the rewarding odor only) were included in the extinction training when using the differential training protocol, whereas all bees were included in extinction training for the absolute conditioning. Therefore, the training curves for extinction training in experiments with differential conditioning start with all bees responding, while those using absolute conditioning start with the same proportion of responses as seen in the acquisition test.

Testing was immediately followed by an additional four presentations of odors alone, each separated by 2 min (extinction training). Five hours later bees were tested again (odor presentation alone) and proboscis extension recorded (extinction test). This method follows the conditioning paradigm used by Maleszka et al. (2000) and training schedule of Lockett et al. (2010). For molecular experiments, only bees that responded correctly during the acquisition test was used for experiments that used extinction training. Analyzed bees were drawn randomly from a cohort of bees that had gone through the exact same training or drug treatment protocol.

### DNA methylation enzyme activity quantification

To assess the activity of DNA methylation enzymes in individual bee brains, honey bees were chilled to −20°C for 3 min before the central brain (excluding optic lobes and gnathal ganglia) was removed in freshly prepared PBS solution. This procedure was performed 1 h after cocaine delivery. Nuclear proteins were extracted using the EpiQuik^TM^ Nuclear extraction kit (Epigentek Group Inc., Farmingdale, NY) and DNA methylation enzyme activity was then measured using an EpiQuik^TM^ DNMT Activity/Inhibition Assay Ultra Kit (Epigentek Group Inc., Farmingdale, NY), according to the manufacturer instructions. Protein concentration was determined with a Bradford assay (Bio-Rad Laboratories Inc., Hercules, CA), and DNA methylation enzyme activity was estimated as follows:

$$\begin{array}{l} \text{DNA methylation enzyme activity}=\\ 1,000\times \frac{\text{Sample OD}-\text{Blank OD}}{\text{Protein amount}\times \text{Incubation time}} \end{array}$$

### Gene expression analysis

To examine transcription levels of *DNA methyltransferase 3* (*AmDNMT3*, GenBank gene ID: 410798) and *TET dioxygenase* (*AmTET*, GenBank gene ID: 412879 412878) following cocaine treatment and extinction training, whole bees were frozen in liquid nitrogen 1 h after treatment (2 h after extinction training). Heads were partially lyophilized prior to the removal of central brains. Dissected brains were stored at −80°C until RNA extraction with the PureLink® RNA Mini Kit (Ambion®, Life Technologies, Grand Island, NY) following supplied guidelines. Each biological replicate consisted of RNA extracted from a single brain. For each experiment, six replicates were performed for each treatment group. Total RNA was reverse transcribed using SuperScript® III First-Strand Synthesis SuperMix kit (Invitrogen™, Life Technologies, Grand Island, NY). Transcription levels were assessed by quantitative real time polymerase chain reactions (qRT-PCR). We performed 10 μL reactions using SsoAdvanced™ Universal SYBR® Green Supermix (Bio-Rad Laboratories Inc., Hercules, CA). Primers used for *AmDNMT3* were: forward: 5′-GAACTCGTTGAAGCAAGGCA-3′; reverse: 5′-AACGTTTGCACGCTCCAAGA-3′, and for *AmTET*: forward 5′-GTCAGTGAGATCAGAGGAGC-3′; reverse 5′-TGGTGCAAGGCTGAGGTACA-3′. The housekeeping genes *AmUGT* (*uridine 5*′*-diphospho-glucuronosyltransferase*) and *AmeIF.S8* (*eukaryotic initiation factor S8)* were used as controls (*AmUGT*, GenBank gene ID: 412198, forwards primer: 5′-CGTTGATGCTGATCAGGTTG-3′; reverse primer: 5′-CGTCGAAATCGCTTCAAGTC-3′; *AmeIF.S8*, GenBank gene ID: 551184, forwards primer: 5′-TGAGTGTCTGCTATGGATTG CAA-3′; reverse primer: 5′-TCGCGGCTCGTGGTAAA-3′). All primers crossed an intron junction in order to avoid potential problems with contamination from genomic DNA, and have previously been used successfully (Foret et al., 2012; Wojciechowski et al., 2014).

### Specimen preparation for quantification of cocaine in honey bee brains by liquid chromatography-mass spectrometry/mass spectrometry

Bees were treated with cocaine in the same manner as for behavioral experiments and placed in an incubator for 15, 30 min, 1, 4, or 24 h, after which central brains were extracted and stored at −80°C until analysis. For analysis brains were resuspended in 60 μL pH 6.0 phosphate buffer and lysed by sonication. Cellular debris was collected by centrifugation at 12,000 g for 10 min at 4°C and the supernatant was removed for analysis. A separate set of untreated brains were processed as above (*n* = 3–6 for each concentration point) and spiked with concentrations of cocaine ranging from 1 to 0.1 ng/mL. These served as a standard for quantification.

### LC-MS/MS

Liquid chromatography-mass spectrometry/mass spectrometry (LC-MS/MS) experiments were performed with an AB SCIEX QTRAP 5500 (SCIEX, Framingham, MA, USA) triple quadrupole linear ion trap mass spectrometer. The LC system used was an Agilent 1100 (Agilent, Santa Clara, CA, USA). Analyst® TF software (version 1.6.2) was used for acquisition and quantitation. Chromatographic separation was achieved using a Zorbex SB-C18 column (150 × 0.5 mm) (Agilent). Elution was performed isocratically with 35% methanol/65% (0.1% formic acid) in H_2_O at a flow rate of 30 μL/min for 8 min total run time. Ten microliter injection volume was used.

MS data was collected in positive ion mode. For quantification and validation of cocaine, the following transitions were monitored, as previously described by Shakleya and Huestis (2009) m/z 304 to 182 (quantification) and m/z 304 to 82 (validation). Peak areas were measured for quantification of each sample.

### Statistical tests

To determine if the cocaine treatment had any effect on learning a χ^2^-test was conducted for each of the two tests (acquisition and extinction). For acquisition and extinction curves a χ^2^-test was used for each point in the curve, adjusted using Bonferroni's correction to account for multiple testing. Effect sizes were estimated using Pearson's φ. Mann-Whitney U-test was used to compare DNA methylation enzyme activity between cocaine and control treated bees. The effect size was estimated using rank biserial correlations (*r*). Transcript levels were compared by *t*-tests, with effect sizes given as Cohen's *d*. All statistical analysis were conducted in R 3.4.1 (R Development Core Team, 2017).

### Experimental order

In order to examine the effects of cocaine on learning, independently of its effects on reward perception, we treated bees with cocaine at various time points before and after acquisition and extinction training. Since we found that cocaine most strongly inhibited consolidation of extinction memory (see below), we tried testing for acquisition memory at the same time point to ensure the effect of cocaine was specific to consolidation of extinction memory. In all of the experiments so far, we used a differential conditioning paradigm. In this paradigm bees are exposed to both aversive and appetitive conditioning. To ensure that the effect of cocaine on consolidation on extinction memory was not the result of an interaction between the processing of these two distinct kinds of memories we repeated the whole conditioning procedure, treating bees 1 h after extinction training, with an absolute conditioning paradigm.

## Results

### Cocaine weakly inhibits acquisition and recall of memory

**Figure 1:**
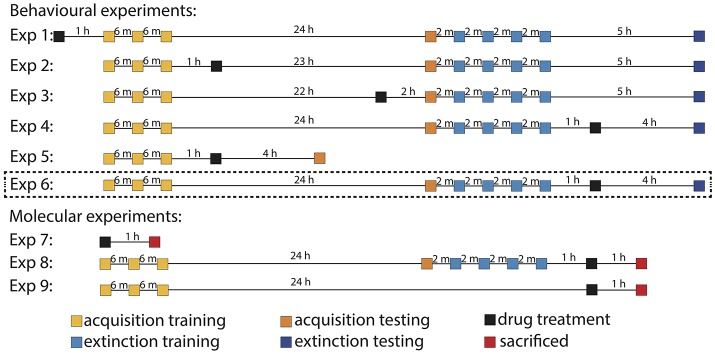
Schematic of training schedules and treatment regimens. Acquisition and extinction trials are presented in yellow and blue, respectively. The darker shades represent test of the same type of memory. The black blocks signify when drug treatment was given, while red blocks show when animals were sacrificed. Note that time is not presented as a linear scale in diagram (h, hours; m, minutes). Dashed lines denote absolute conditioning was used instead of differential.

**Figure 2:**
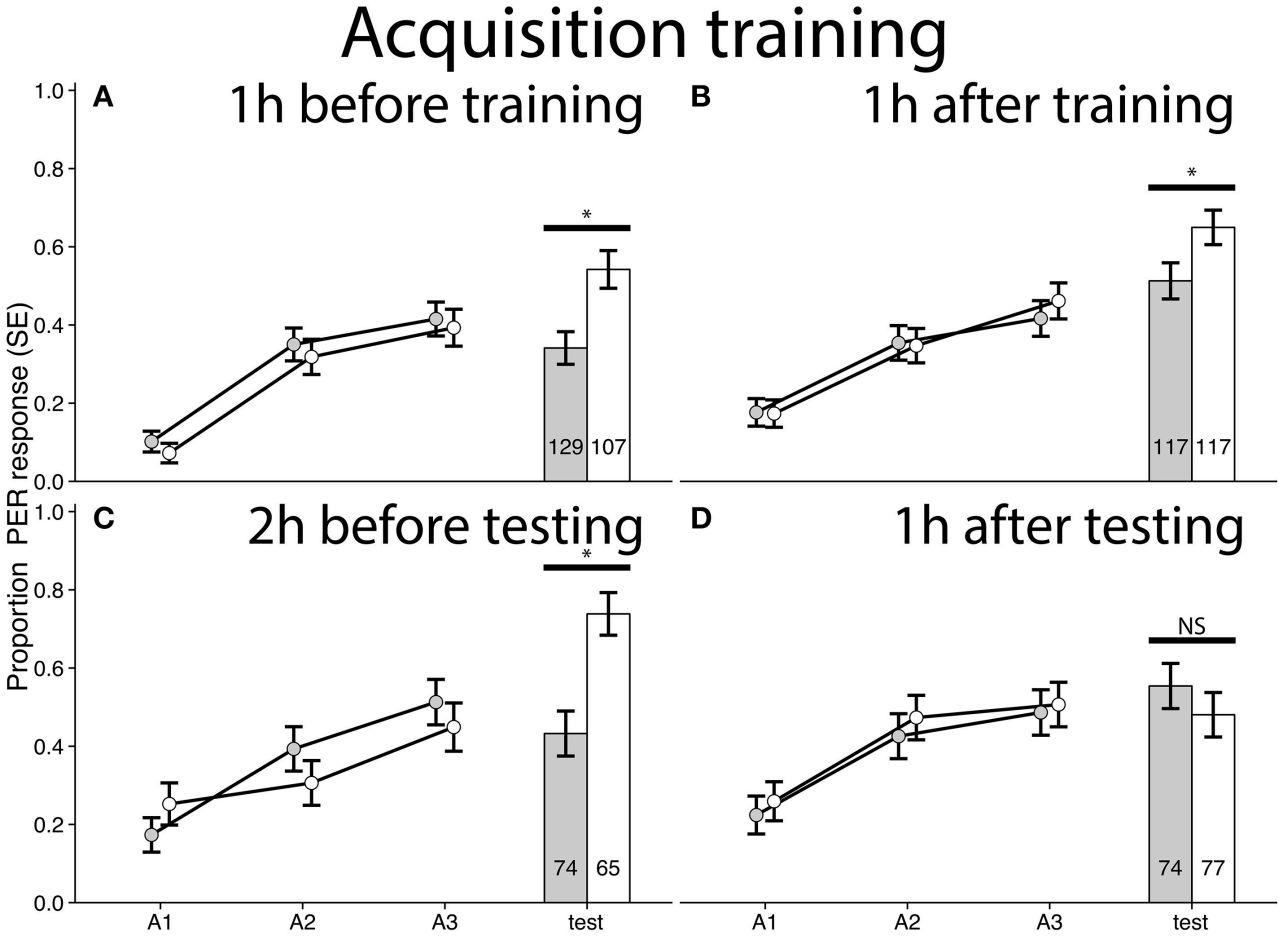
Acquisition curves and test results for bees trained in a differential conditioning PER training paradigm. A1–A3 refers to each odor exposure during conditioning. Gray bars/dots represent bees treated with cocaine and controls are in white. Responses shown for the odor paired with sucrose reward, see Figure S1 for responses of the odor paired with NaCl. **(A)** Experiment 1: Bees treated with cocaine 1 h before training (χ^2^ = 8.8245, *p* = 0.002972, *n* = 236, ϕ = 0.1933). **(B)** Experiment 2: Bees treated 1 h after training (χ^2^ = 3.9503, *p* = 0.04686, *n* = 234, ϕ = 0.1299). **(C)** Experiment 3: Bees treated 2 h before testing (χ^2^ = 12.043, *p* = 0.0005, *n* = 139, ϕ = 0.2943). **(D)** Experiment 4: Bees treated 1 h after testing (χ^2^ = 0.5491, *p* = 0.4587, *n* = 151). ^*^Denotes statistically significant differences.

**Figure 3:**
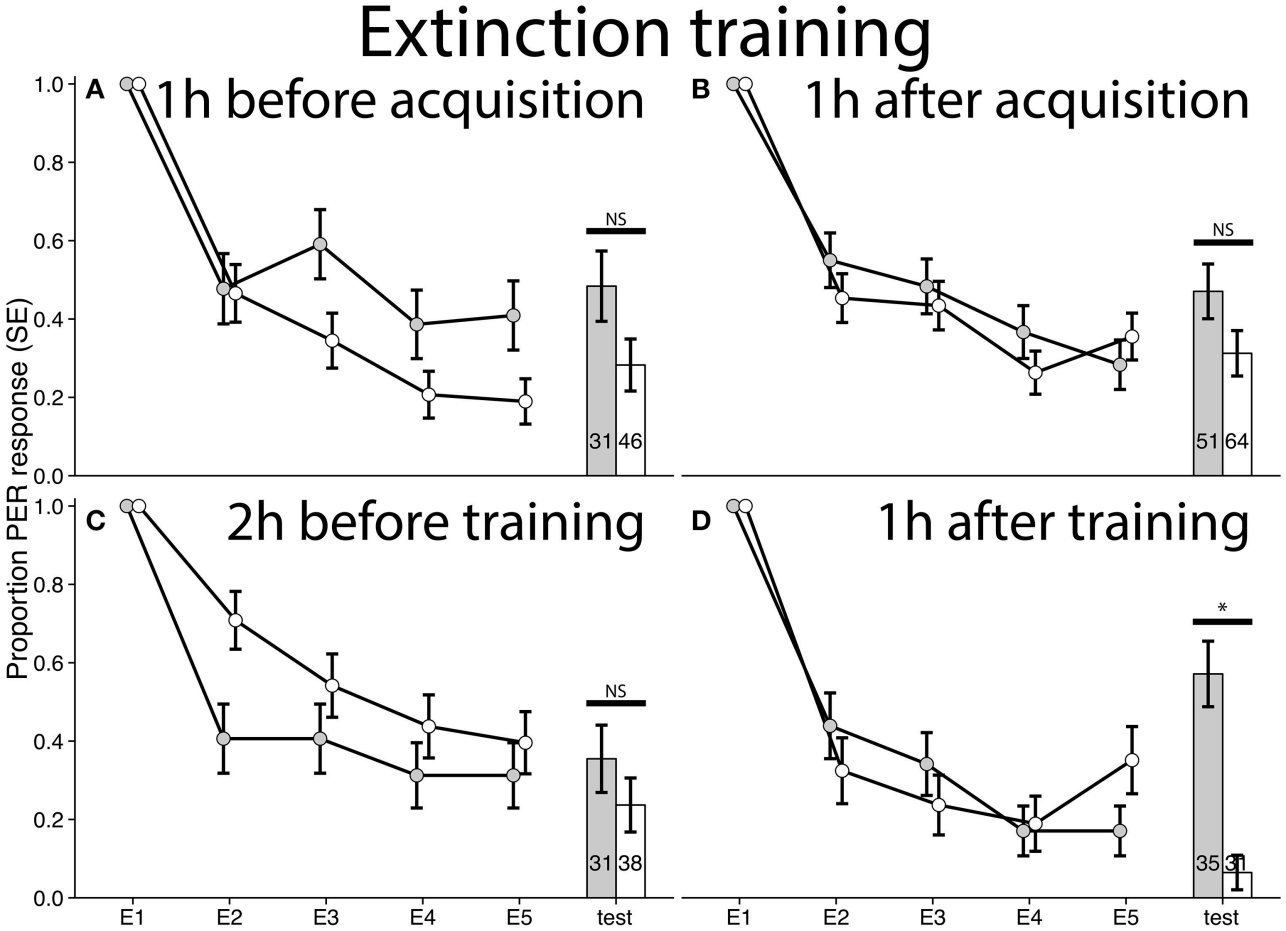
Extinction curves and test results for bees trained in a differential conditioning PER training paradigm. E1–E5 refers to each odor exposure during extinction. Gray bars/dots represent bees treated with cocaine and controls are in white. **(A)** Experiment 1: Bees treated with cocaine 1 h before acquisition training (χ^2^ = 2.4304, *p* = 0.119, *n* = 77). **(B)** Experiment 2: Bees treated 1 h after acquisition training (χ^2^ = 2.3709, *p* = 0.1236, *n* = 115). **(C)** Experiment 3: Bees treated 2 h before extinction training (χ^2^ = 0.6527, *p* = 0.4192, *n* = 69). **(D)** Experiment 4: Bees treated 1 h after extinction training (χ^2^ = 16.7965, *p* < 0.00001, *n* = 66, ϕ = 0.5044). Note that for extinction memory, lack of PER expression signifies successful memory formation. ^*^Denotes statistically significant differences.

Cocaine treatment applied 1 h before training (Figure 1) does not alter the rate of acquisition or extinction of learning of an appetitive memory as evidenced by comparison of acquisition and extinction learning curves for cocaine treated and control bees for any of the experiments (Figures 2, 3). There were, however, differences in the recall of acquisition memory for bees treated with cocaine 1 h before (Experiment 1: χ^2^ = 8.8245, *p* = 0.0030, *n* = 236, ϕ = 0.1933, Figure 2A) and after acquisition training (Experiment 2: χ^2^ = 3.9503, *p* = 0.0469, *n* = 234, ϕ = 0.1299, Figure 2B), and 2 h before the recall of acquisition memory (Experiment 3: χ^2^ = 12.043, *p* = 0.0005, *n* = 139, ϕ = 0.2943, Figure 2C). In other words, bees that had been treated with cocaine prior to the acquisition test (Experiments 1–3), regardless of time point (before conditioning, after conditioning, or before recall) performed worse in the recall of acquisition test than control bees (Figure 2). There was no difference in response rate to the aversive memory for any of the treatment timepoints (Figure S1).

### Cocaine strongly inhibits consolidation of extinction

**Figure 4:**
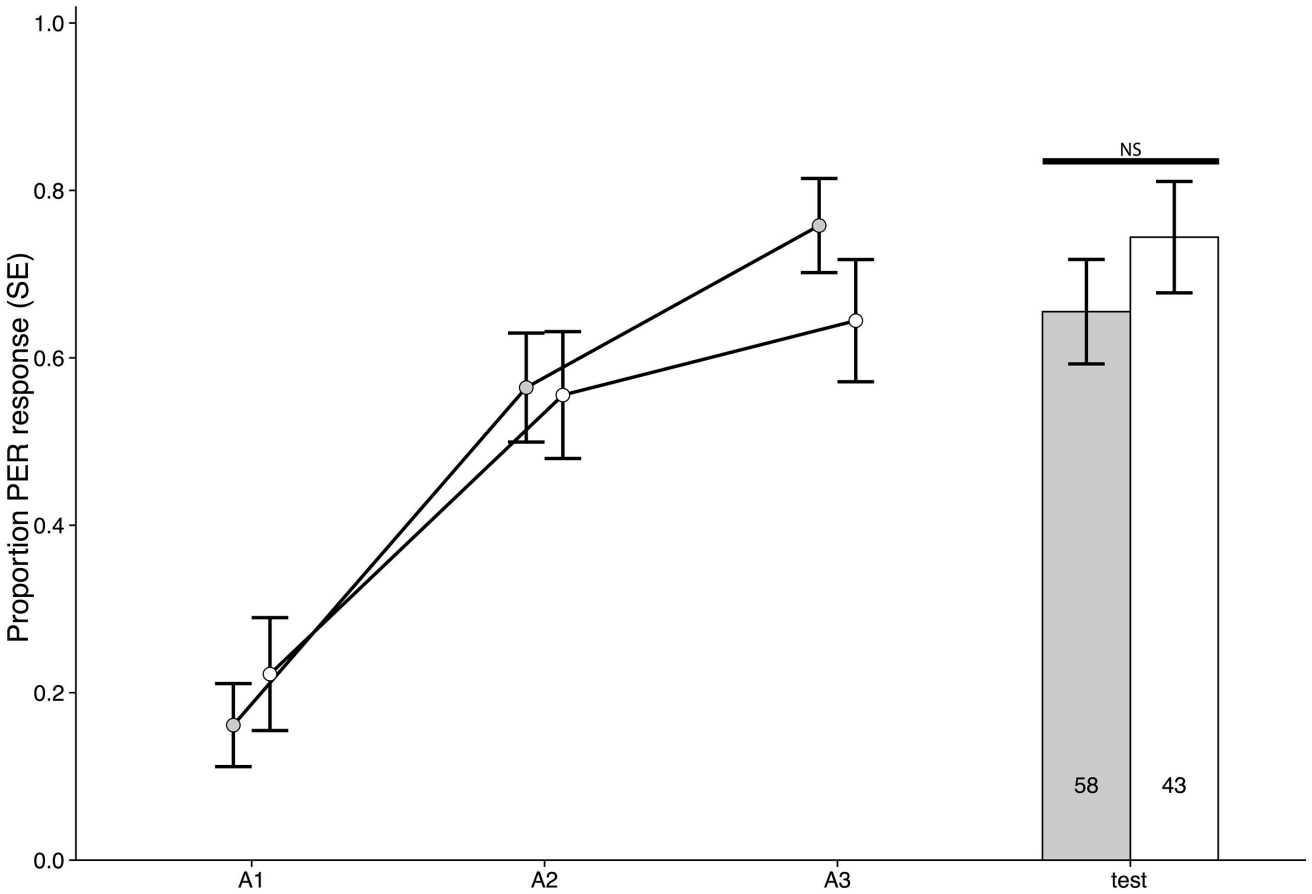
Experiment 5: acquisition curve and test results for bees trained in a differential conditioning PER paradigm and treated with cocaine 1 h after acquisition training, but tested 5 h after training (χ^2^ = 0.5489, *p* = 0.4588, *n* = 101). Responses shown for the odor paired with sucrose reward, see Figure S2 for responses of the odor paired with NaCl.

When we tested the recall of extinction conditioning, however, it was only when cocaine was administered 1 h post-extinction training that there was a difference between cocaine and control groups (Experiment 4: χ^2^ = 16.7965, *p* < 0.00001, *n* = 66, ϕ = 0.5044, Figure 3D). Here, cocaine treatment impaired consolidation of extinction conditioning resulting in a stronger response to the training odor during the extinction test (Figure 3). Because the extinction test was 5 h after conditioning (as opposed to the 24 h gap between acquisition training and testing) we could not be sure if the effect seen was specific to the extinction paradigm or a general performance change 4 h after cocaine treatment. We therefore treated bees with cocaine 1 h after acquisitioning training and tested them 4 h later. We did not detect any difference between treatment and control treated bees in response to odor paired with sucrose (Experiment 5: χ^2^ = 0.5489, *p* = 0.4588, *n* = 101, Figure 4) or NaCl (Figure S2). This suggests the effect seen in Figure 3 is specific to consolidation of extinction memory.

**Figure 5:**
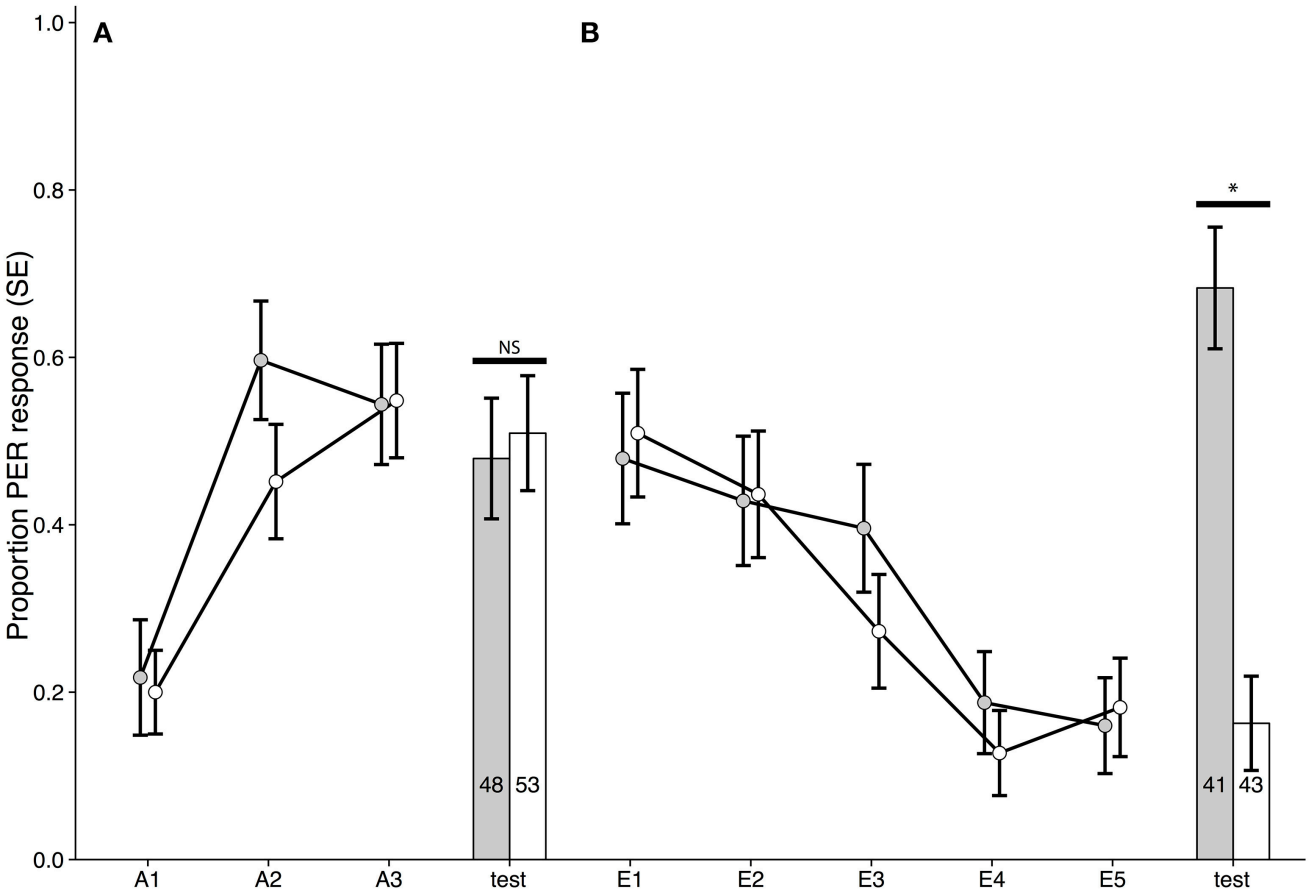
Experiment 6: results for bees trained in a single odor conditioning PER paradigm. Cocaine treatment was given 1 h after extinction training. **(A)** Acquisition test (χ^2^ = 0.0109, *p* = 0.9167, *n* = 101). **(B)** Extinction test (χ^2^ = 21.2706, *p* < 0.000001, *n* = 84, ϕ = 0.5032). Note that for extinction memory, lack of PER expression signifies successful memory formation. ^*^Denotes statistically significant differences.

We further examined the robustness of this phenomenon by training bees in an absolute conditioning paradigm with a rewarded odor only. When cocaine treatment was given 1 h after training the same effect was seen (Experiment 6: χ^2^ = 21.2706, *p* < 0.000001, *n* = 84, ϕ = 0.5032, Figure 5). Thus, we conclude that cocaine has a strong inhibitory effect on consolidation of extinction memory.

### Cocaine affects DNA methylation dynamics

**Figure 6:**
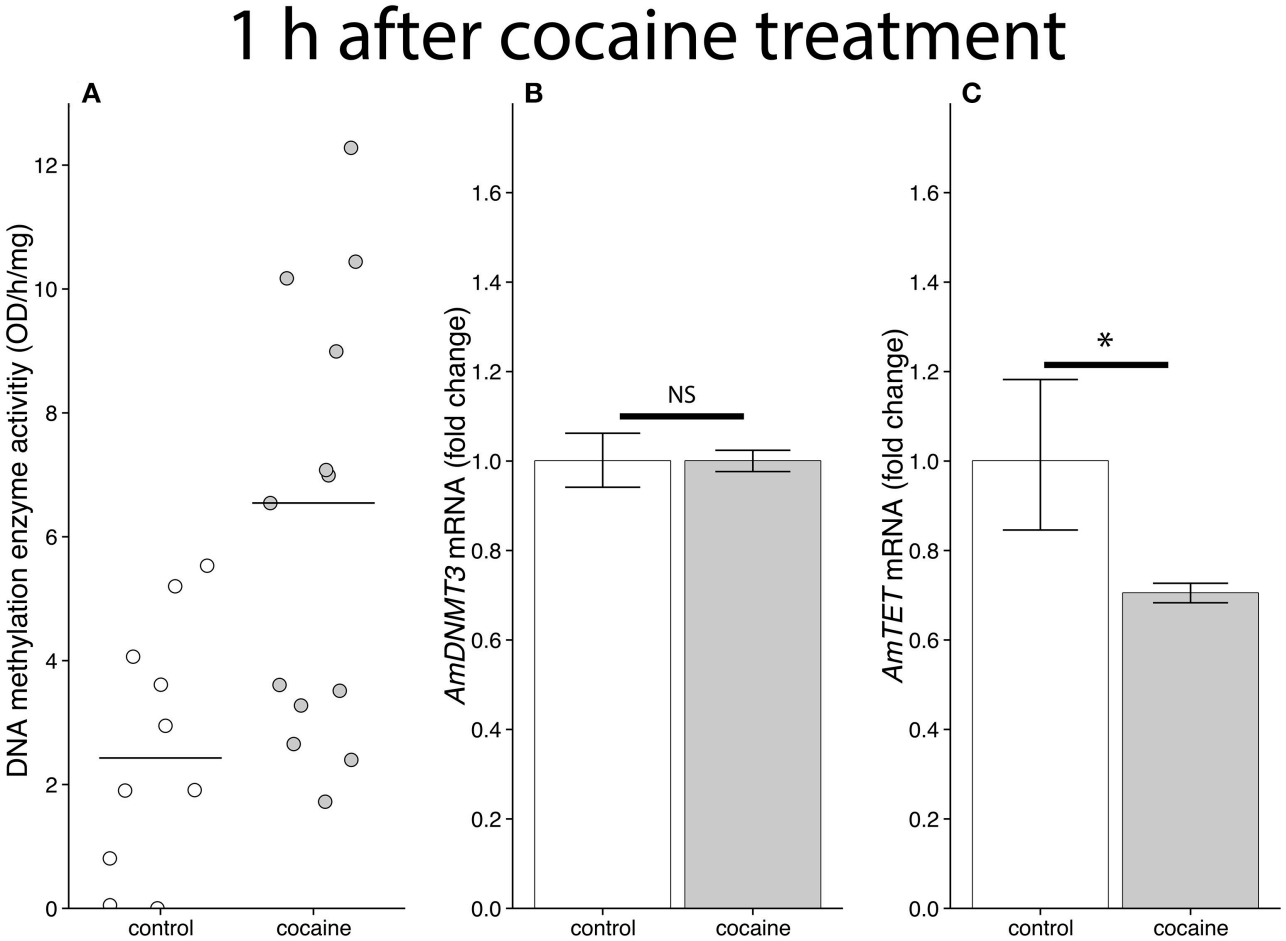
The effects of cocaine on DNA methylation enzyme activity and *AmDNMT3* and *AmTET* and transcription levels, 1 h after cocaine treatment. **(A)** DNA methylation enzyme activity following cocaine administration is displayed as a function of relative optic density. Black lines show median values, dots represent values for samples consisting of four pooled brains. DNMT activity was significantly increased following exposure to 3 μg of cocaine (Mann-Whitney *U* = 29, *p* = 0.01276, *r* = 0.4994). **(B)** There was no significant difference in *AmDNMT3* mRNA levels following cocaine or control treatments (*AmUGT*: t_10_ = 1.3439, *p* = 0.1940; *AmeIF.S8*: t_10_ = 0.0001, *p* = 0.9999). **(C)** There was a significant reduction in levels of *AmTET* mRNA in bees treated with cocaine (*AmUGT:* t_10_ = −5.0172, *p* < 0.0001, *d* = −1.5866; *Ame.IF.S8*: t_10_ = −5.3780, *p* < 0.0001, *d* = −1.7007). ^*^Denotes statistically significant differences.

DNA methylation enzyme activity was increased in honey bee brains 1 h following cocaine administration (Experiment 7: Mann-Whitney *U* = 29, *p* = 0.01276, *r* = 0.4994, Figure 6A). Cocaine treatment did not affect transcription levels of *AmDNMT3*, which is believed to be responsible for *de novo* methylation in honey bees (Wang et al., 2006), in bees 1 h after cocaine treatment (Experiment 7: *AmUGT*: t_10_ = 1.3439, *p* = 0.1940; *AmeIF.S8*: t_10_ = 0.0001, *p* = 0.9999; Figure 6B). We next examined the effects of cocaine treatment and associative learning on levels of the honey bee homolog of TET dioxygenase, which has been shown to be responsible for removal of methylation marks in honey bees (Wojciechowski et al., 2014). Cocaine treatment caused a significant decrease in transcript levels of *AmTET* (Experiment 7: *AmUGT*: t_10_ = −5.0172, *p* < 0.0001, *d* = −1.5866; *Ame.IF.S8*: t_10_ = −5.3780, *p* < 0.0001, *d* = −1.7007; Figure 6C).

### The effects of cocaine on TET is context dependent

**Figure 7:**
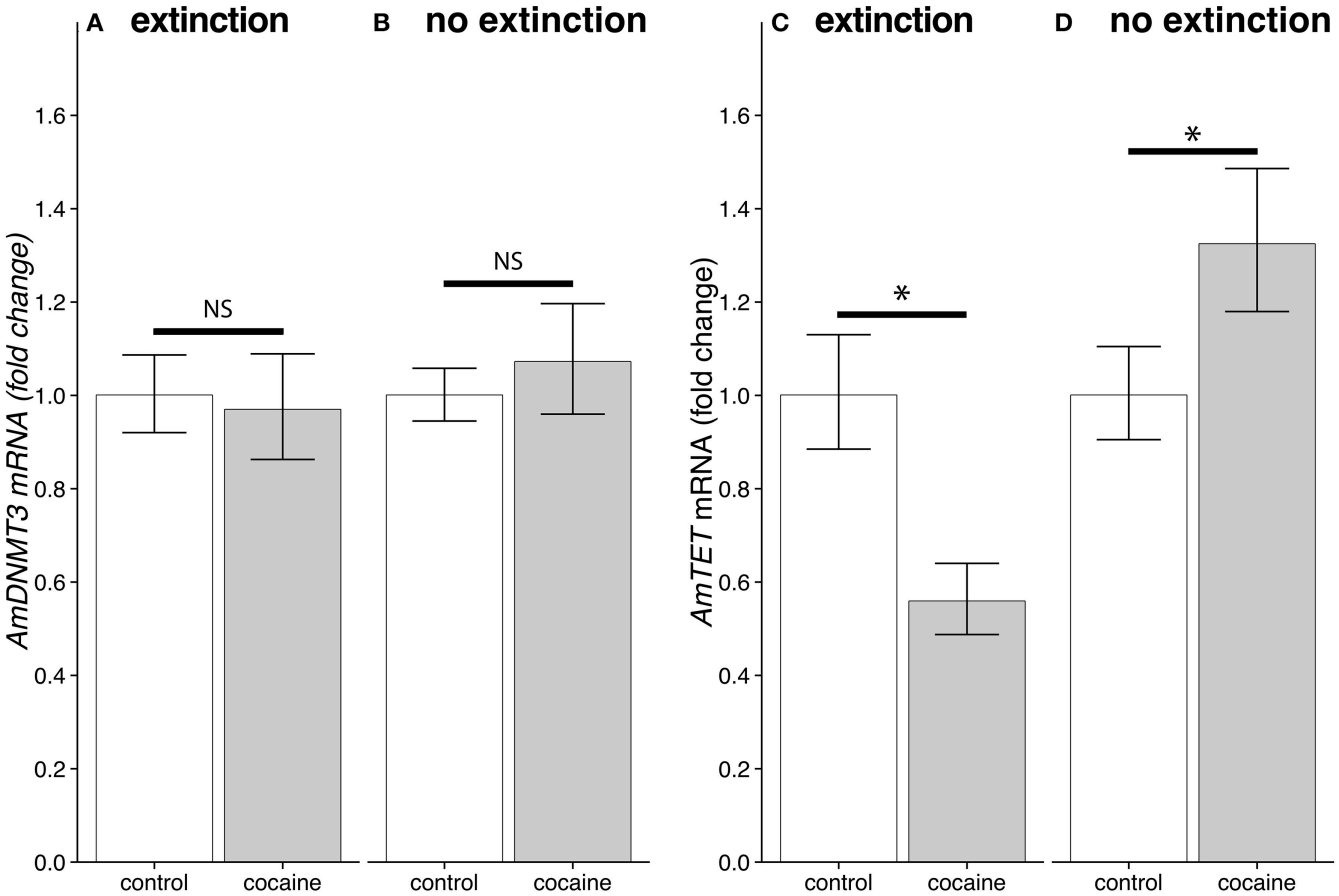
The effects of cocaine and extinction training on levels of DNA methylation enzymes 1 h after treatment. **(A)** There was no significant difference in *AmDNMT3* mRNA levels following cocaine or control treatments given after extinction training (*AmUGT*: t_10_ = 0.8532, *p* = 0.4135; *AmeIF.S8*: t_10_ = −0.6977, *p* = 0.4927). **(B)** Similarly, levels were not affected by cocaine in bees that did not go through extinction training (*AmUGT*: t_10_ = 0.9113, *p* = 0.3836; *AmeIF.S8*: t_10_ = 1.8837, *p* = 0.0729). **(C)** There was a significant reduction in levels of *AmTET* mRNA in bees treated with cocaine following extinction training (*AmUGT*: t_10_ = −3.6832, *p* = 0.0013, *d* = −1.1105; *AmeIF.S8*: t_10_ = −7.8450, *p* < 0.0001, *d* = −2.3653). **(D)** Intriguingly, bees that only went through acquisition training, prior to being treated with cocaine showed the opposite effect (*AmUGT*: t_10_ = 14.1652, *p* < 0.0001, *d* = 4.2710; *AmeIF.S8*: t_10_ = 6.9097, *p* < 0.0001, *d* = 2.0834). ^*^Denotes statistically significant differences.

The effects of cocaine on *AmTET*, but not *AmDNMT3*, expression levels varied with the learning experience of the bees. Levels of *AmDNMT3* mRNA were not affected by cocaine in bees that had gone through extinction conditioning (Experiment 8: *AmUGT*: t_10_ = 0.8532, *p* = 0.4135; *AmeIF.S8*: t_10_ = −0.6977, *p* = 0.4927; Figure 7A). Cocaine treatment did not alter *AmDNMT3* levels in bees that had not gone through extinction training either (Experiment 9: *AmUGT*: t_10_ = 0.9113, *p* = 0.3836; *AmeIF.S8*: t_10_ = 1.8837, *p* = 0.0729; Figure 7B). In contrast, levels of *AmTET* were significantly reduced following cocaine treatment after extinction conditioning (Experiment 8: *AmUGT*: t_10_ = −3.6832, *p* = 0.0013, *d* = −1.1105; *AmeIF.S8*: t_10_ = −7.8450, *p* < 0.0001, *d* = −2.3653; Figure 7C). Intriguingly, when bees that were treated with cocaine after acquisition training *AmTET* levels increased significantly (Experiment 9: *AmUGT*: t_10_ = 14.1652, *p* < 0.0001, *d* = 4.2710; *AmeIF.S8*: t_10_ = 6.9097, *p* < 0.0001, *d* = 2.0834; Figure 7D). Taken together, these results suggest that the effects of cocaine on *AmTET* levels were dependent on the learning experience of the bees.

### Retention of cocaine post-treatment

**Figure 8:**
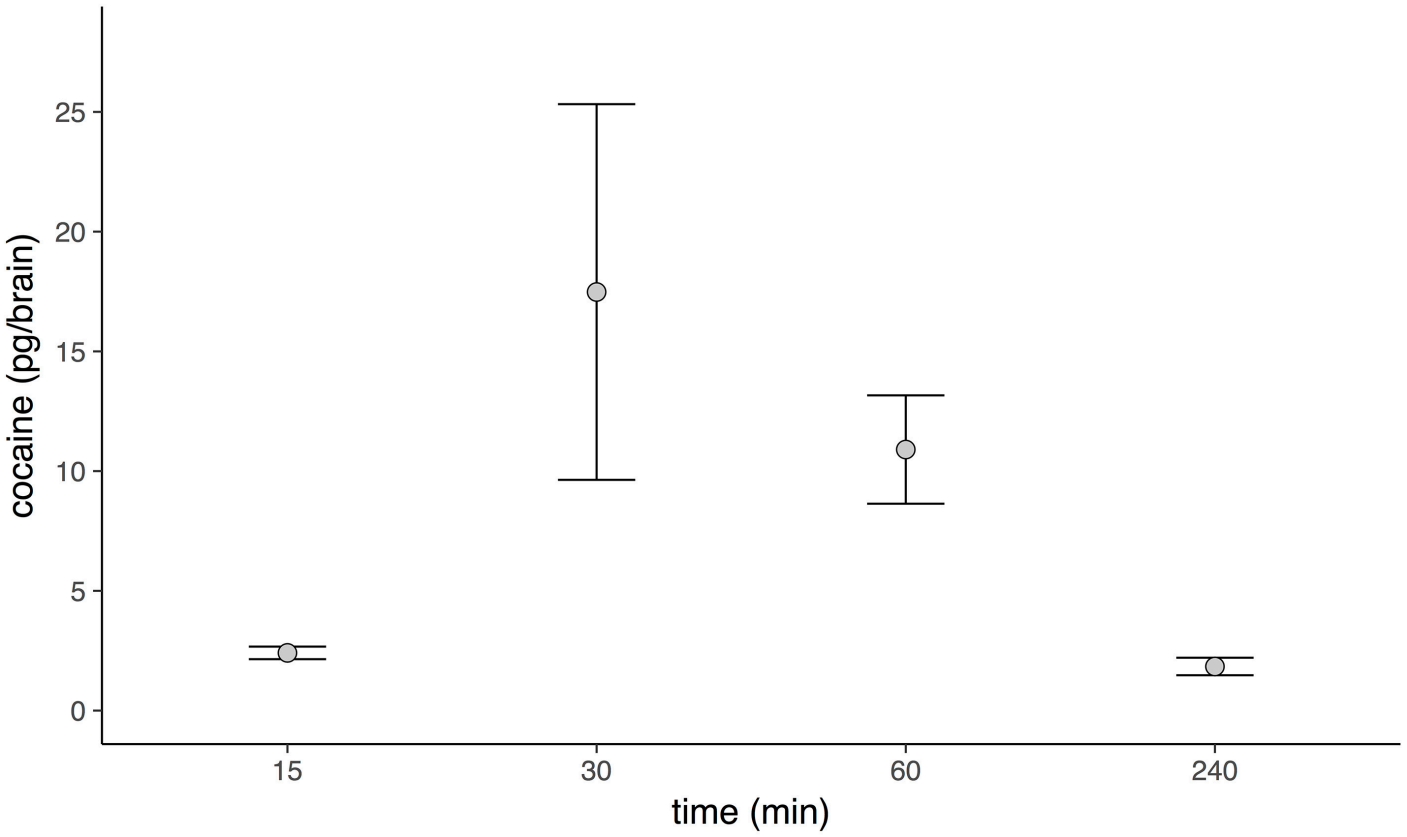
Amounts of cocaine in the honey bee brain after topical treatment. Levels of cocaine increased sharply 30 min after treatment, thereafter levels gradually decreased, until they were almost absent at 4 h.

The total amounts of cocaine present in brains peaked 30 min after treatment and gradually declined to almost zero over a 4 h period (Figure 8).

## Discussion

Our experiments show a far stronger effect of cocaine on consolidation of extinction memory (Figures 3D, 5) than consolidation of acquisition memory (Figures 2B, 4), as evidenced by the difference in effect size between these experiments. Cocaine treatment affects performance in the recall of acquisition memory when it was applied before testing (Figure 2C). Treatment prior to acquisition training does not affect the rate of acquisition, but still impair memory formation (Figure 2A). Cocaine did not affect naïve odor responses as no difference was seen between treatment groups on the first exposure to odors in any of our experiments where cocaine was administered prior to acquisition training.

The lack of any effect of cocaine on rate of learning (demonstrated by the overlapping acquisition and extinction curves of the two experimental groups, Figures 2–4) was expected, as we intentionally chose time points for cocaine administration that would not cause cocaine to interfere with perception of stimuli during acquisition and extinction conditioning. This design allowed us to examine any direct effects of cocaine on learning and memory, rather than the effects of changed incentive salience for the conditioned stimuli. The strongest effect of cocaine, seen in this context, was an impairment of the consolidation of extinction memory (Figures 3D, 5). This suggests that part of the reason why cocaine-associated memories are so hard to extinguish, could be that in addition to increasing the incentive salience of stimuli (Uslaner et al., 2006), cocaine also actively inhibits consolidation of extinction. A likely mechanistic explanation for this phenomenon could be that cocaine interferes with the epigenetic mechanisms of memory consolidation (Day and Sweatt, 2010; Robison and Nestler, 2011).

There is ample evidence from the mammalian literature that chronic cocaine administration interferes with DNA methylation dynamics (Robison and Nestler, 2011). In mice levels of *DNMT3a*, but not *DNMT1* and *DNMT3b*, increase following chronic cocaine exposure (LaPlant et al., 2010). This increase persist for at least 28 days after the end of drug treatments (LaPlant et al., 2010). The altered levels of *DNMT3a* have been shown to affect the DNA methylation patterns in the brains of mice following cocaine exposure (Anier et al., 2010). Further, Feng et al. (2015) has shown that levels of *TET* are also decrease after chronic cocaine exposure. The effects on both *DNMT3a* and *TET* have been shown to be localized to particular brain regions and affect the methylation and demethylation of specific genomic regions (LaPlant et al., 2010; Feng et al., 2015). Thus, it is becoming increasingly clear that changes to brain DNA methylation dynamics play a role in the behavioral outcomes associated with cocaine abuse.

In this study we show that in bees a single cocaine treatment can alter DNA methylation enzyme activity levels (Figure 6A) and transcription of *AmTET* (Figures 6C, 7C,D). So far the results for *AmTET* largely mimics what is seen in mammalian systems, however, unlike studies in mice, we failed to detect any change in *AmDNMT3* levels in both naïve and trained bees. The most likely explanation for this difference is that mice were treated chronically over the course of 28 days, while the bees in our study received a single cocaine treatment. It is of course possible that *AmDNMT3* levels are affected in bees following chronic exposure.

Another possibility is that *AmDNMT3* is not a primary *de novo* methyltransferase in honey bees (Wedd and Maleszka, 2016). Recent studies suggest that the classical roles of DNMT1 and DNMT3s in establishing methylation patterns need to be redefined to include the evident *de novo* activity of DNMT1 and DNMT3s' involvement in maintenance (Jeltsch and Jurkowska, 2014). The variation of DNMTs across invertebrates is also suggestive of diverse roles for these enzymes. In the honey bee, the DNA methylation toolkit consists of two copies of DNMT1 and one copy of DNMT3, but many insects lack DNMT3 and still methylate their genome implying that DNMT1 provides *de novo* activity in these organisms (Wedd and Maleszka, 2016).

Perhaps the most surprising result is the context dependency of the effects of cocaine on *AmTET* levels. We can only speculate why levels were shown to increase in animals following acquisition conditioning, while they were decreased in naïve animals and animals that had gone through extinction training, but it is a clear demonstration that when considering how drugs of abuse might interact with the DNA methylation/demethylation machinery it is important to remember that what the animal is actively doing or exposed to can play an important role. A further issue that must be taken seriously is where in the brain the enzymes responsible, for methylation and demethylation, act during memory formation and where cocaine is exerting its effects. In this study we used a systemic cocaine treatment and all analysis of gene expression or enzyme activity occur at the whole-brain level, we are thus not able to conclude anything clearly about this question. Biergans et al. (2017) beautifully demonstrated how DNMT activity in the antennal lobes mediates odor specificity during learning. Another interesting to note is that many of the methylation related genes shown to have altered expression patterns following olfactory conditioning are highly expressed in the honey bee mushroom bodies (Biergans et al., 2015). We therefore think that the antennal lobe and mushroom bodies are key structures for future investigations.

The finding that cocaine interferes with consolidation of extinction learning, potentially by changing levels of *AmTET*, has implications for the application of extinction-based therapies. While this method has worked well when attempting to extinguish fearful memories (Schiller et al., 2010), it has been less successful for treating addiction (Conklin and Tiffany, 2002), as marked by very high rates of relapse in recovering addicts (McLellan et al., 2000; Hser et al., 2001). If cocaine uniquely interferes with mechanisms involved in consolidation of extinction memory, it could potentially mean that extinction therapies are severely compromised in recovering addicts who still occasionally use cocaine.

## Author contributions

ES, AB, and RM conceived the study. ES, PD, WK, PH, EB, and JP performed the experiments. ES analyzed the data. ES, AB, RM, PD, WK, PH, EB, and JP wrote the manuscript.

### Conflict of interest statement

The authors declare that the research was conducted in the absence of any commercial or financial relationships that could be construed as a potential conflict of interest.
