# Supplementary material for: Cocaine Directly Impairs Memory Extinction and Alters Brain DNA Methylation Dynamics in Honey Bees
Source: Front Physiol. 2018 Feb 13;9:79. doi: 10.3389/fphys.2018.00079 (PMC5816933; doi:10.3389/fphys.2018.00079)
Supplement: Supplementary file 1 [file Image1.PDF]

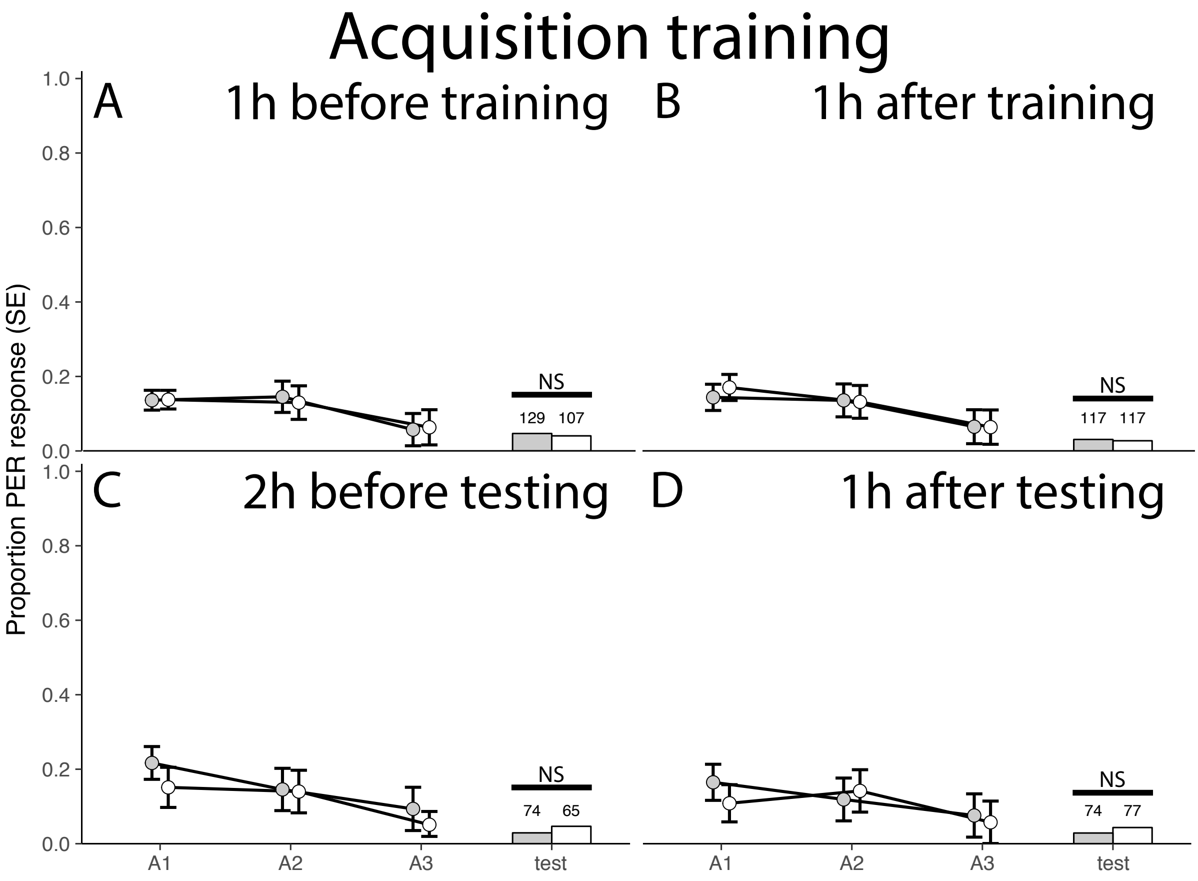

Figure S1. Acquisition curves and test results for odour paired with aversive stimuli for bees trained in a differential conditioning PER training paradigm. A1-A3 refers to each odour exposure during conditioning. Grey bars/dots represent bees treated with cocaine and controls are in white. Responses to the odour paired with sucrose reward are shown in Figure 2. **A.** Experiment 1: Bees treated with cocaine 1 hour before training ($\chi^2 < 0.0001$, p = 1.0, n = 236). **B.** Experiment 2: Bees treated one hour after training ($\chi^2 < 0.0001$, p = 1.0, n = 234). **C.** Experiment 3: Bees treated 2 hours before testing ($\chi^2 = 0.0095$, p = 0.9223, n = 139). **D.** Experiment 4: Bees treated one hour after testing ($\chi^2 = 0.0023$, p = 0.9619, n = 151).

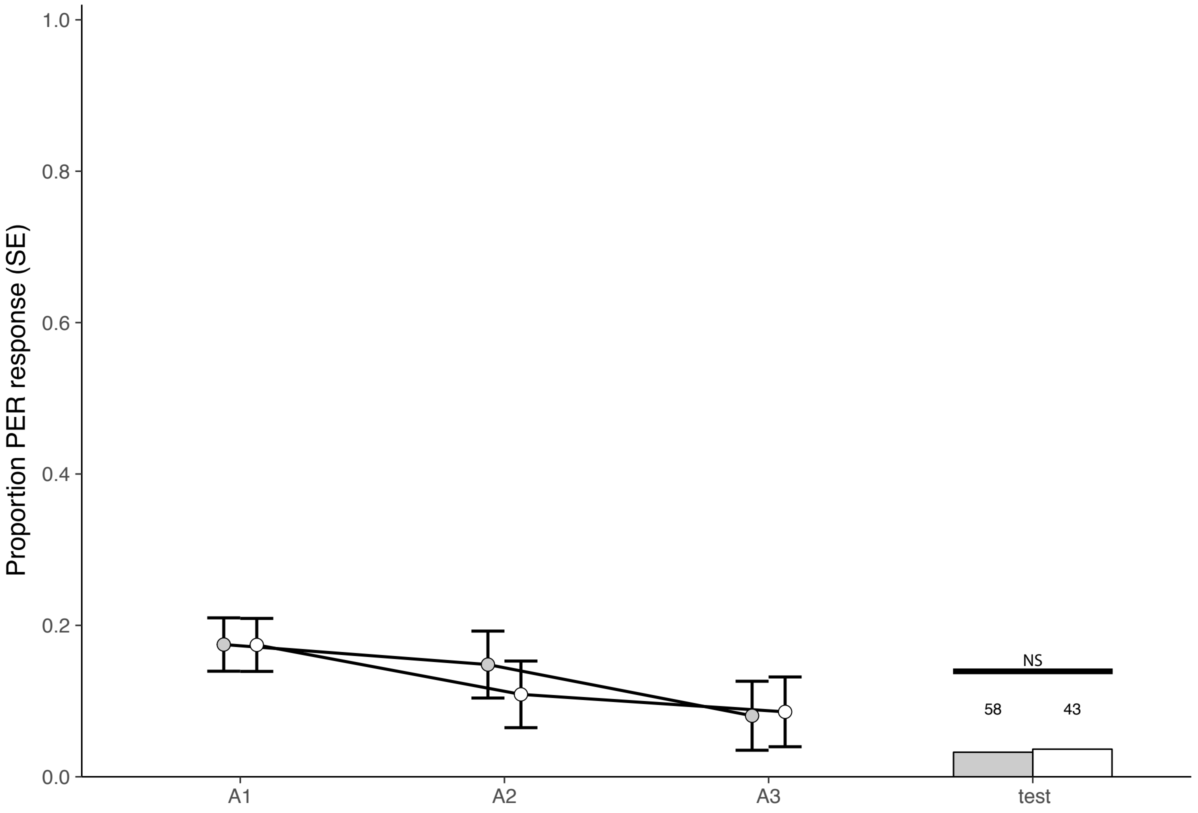

Figure S2. Experiment 5: acquisition curve and test results for odour paired with aversive stimuli for bees trained in a differential conditioning PER training paradigm and treated with cocaine 1 hour after acquisition training, but tested 5 hours after training ($\chi^2 < 0.0001$, p = 1.0, n = 101). Responses to the odour paired with sucrose reward are shown in Figure 4.
